# Mitochondrial *Arabidopsis thaliana* TRXo Isoforms Bind an Iron–Sulfur Cluster and Reduce NFU Proteins In Vitro

**DOI:** 10.3390/antiox7100142

**Published:** 2018-10-13

**Authors:** Flavien Zannini, Thomas Roret, Jonathan Przybyla-Toscano, Tiphaine Dhalleine, Nicolas Rouhier, Jérémy Couturier

**Affiliations:** 1 Université de Lorraine, Inra, IAM, F-54000 Nancy, France; flavien.zannini@univ-lorraine.fr (F.Z.); thomas.roret@sb-roscoff.fr (T.R.); przybylajonathan@orange.fr (J.P.-T.); tiphaine.dhalleine@univ-lorraine.fr (T.D.); nicolas.rouhier@univ-lorraine.fr (N.R.); 2 CNRS, LBI2M, Sorbonne Universités, F-29680 Roscoff, France; 3 Department of Plant Physiology, Umeå Plant Science Centre, Umeå University, S-90187 Umea, Sweden

**Keywords:** mitochondria, thioredoxin, iron–sulfur cluster, redox regulation

## Abstract

In plants, the mitochondrial thioredoxin (TRX) system generally comprises only one or two isoforms belonging to the TRX h or o classes, being less well developed compared to the numerous isoforms found in chloroplasts. Unlike most other plant species, *Arabidopsis thaliana* possesses two TRXo isoforms whose physiological functions remain unclear. Here, we performed a structure–function analysis to unravel the respective properties of the duplicated TRXo1 and TRXo2 isoforms. Surprisingly, when expressed in *Escherichia coli*, both recombinant proteins existed in an apo-monomeric form and in a homodimeric iron–sulfur (Fe-S) cluster-bridged form. In TRXo2, the [4Fe-4S] cluster is likely ligated in by the usual catalytic cysteines present in the conserved Trp-Cys-Gly-Pro-Cys signature. Solving the three-dimensional structure of both TRXo apo-forms pointed to marked differences in the surface charge distribution, notably in some area usually participating to protein–protein interactions with partners. However, we could not detect a difference in their capacity to reduce nitrogen-fixation-subunit-U (NFU)-like proteins, NFU4 or NFU5, two proteins participating in the maturation of certain mitochondrial Fe-S proteins and previously isolated as putative TRXo1 partners. Altogether, these results suggest that a novel regulation mechanism may prevail for mitochondrial TRXs o, possibly existing as a redox-inactive Fe-S cluster-bound form that could be rapidly converted in a redox-active form upon cluster degradation in specific physiological conditions.

## 1. Introduction

Mitochondria are important organelles being notably the site of production of cellular energy in the form of adenosine triphosphate (ATP) through the process of oxidative phosphorylation and being also an important site for the amino acid and lipid metabolisms and for the biosynthesis of many crucial vitamins and cofactors. For instance, mitochondria are crucial for the synthesis of the iron–sulfur (Fe-S) clusters found in numerous essential proteins present in the matrix but also in the cytosol and the nucleus [1,2,3]. Despite the existence of several energy-dissipating systems, the over-reduction of the mitochondrial electron transport chain (ETC) releases reactive oxygen species (ROS) notably at the level of the complexes I and III [4]. Scavenging enzymes for the superoxide ion and H_2_O_2_ are present in the mitochondrial matrix. This includes superoxide dismutases and ascorbate- and thiol-dependent peroxidases belonging to the peroxiredoxin (PRX) or glutathione-peroxidase-like (GPXL) families [4]. These thiol peroxidases use reactive cysteine residues for catalysis and rely either on a glutathione/glutaredoxin (GSH/GRX) system or on a thioredoxin (TRX) system for their regeneration [5,6,7,8]. Despite the existence of these scavenging systems, ROS-mediated protein oxidation occurs in this compartment. At the level of some sensitive protein cysteine residues, this leads to reversible modifications such as the formation of sulfenic acid, of disulfide bonds, or of glutathionylated or nitrosylated cysteines. In this respect, it is surprising that the systems devoted to the reduction of these oxidized cysteine forms are not very developed in mitochondria. Among the 30 genes coding for GRXs [9] and the 30 to 45 genes encoding TRX or TRX-like proteins in angiosperms [10,11], only one GRX named GRXS15 [12] and one or two TRXs are found in mitochondria, the majority being present in plastids and in the cytosol/nucleus. The mitochondrial TRX isoforms belong to the TRXh or TRXo classes [13,14]. Unlike most other plant species, *A. thaliana* possesses two TRXo isoforms which add to TRXh2 [15]. Their regeneration should be in principle dependent on the nicotinamide adenine dinucleotide phosphate reduced (NADPH)-thioredoxin reductase (NTR) A and/or B [13,16]. Concerning GRXS15, no in vitro GSH-dependent reductase activity was observed for GRXS15 so far whereas it possesses an oxidase activity being able to oxidize the reduction-oxidation sensitive green fluorescent protein 2 (roGFP2) at the expense of oxidized glutathione (GSSG) [17]. The physiological relevance of this activity is unclear because it is rather thought that GRXS15 participates to the maturation of Fe-S proteins serving as a transfer protein for the exchange of Fe-S clusters from scaffold proteins, on which de novo synthesis occurs, either to other maturation factors or directly to acceptor proteins [12,18]. Hence, TRXs o and h likely represent the major disulfide reductases in plant mitochondria. 

Both *A. thaliana* TRXs o have the typical characteristics of TRXs i.e., a molecular weight around 13 kDa and a conserved WCGPC motif that comprises the catalytic cysteines. In their reduced forms, TRXs are rather competent for reducing disulfide bonds in target proteins. Nevertheless, as demonstrated for some peculiar TRXs, they might also reduce S-nitrosylated or S-glutathionylated proteins [19,20]. If TRXs o also possessed such a property, this would help understanding the absence of GRXs with deglutathionylation activity in mitochondria. The molecular function of these TRXs o is unclear and only *A. thaliana* TRXo1 and pea TRXo were studied so far. TRXs o may function in the regeneration of peroxiredoxin IIF [7,8], in the activation by reduction of citrate synthase [21], alternative oxidase (AOX) [22,23], and isocitrate dehydrogenase [24], but also in the deactivation of both mitochondrial succinate dehydrogenase and fumarase [25]. However, at the macromolecular level, the phenotypes of *A. thaliana* mutants for *trxo1* and *trxo2* single mutants or for the double mutant are extremely mild. In one study, no growth defect was observed for these mutants grown on soil under long-day conditions for four weeks [26]. In other studies, the *A. thaliana trxo1* mutant showed an accelerated germination in the presence of salt [27], and a significant reduction in the fresh weight of shoots was visible during the first four weeks of growth whereas the root growth was not affected [25]. At the cellular level, the activity of enzymes of the tricarboxylic acid (TCA) cycle, or associated with it, is deregulated is the *A. thaliana trxo1* mutant and this is accompanied by changes in the amounts of some metabolites, notably citrate, malate, and pyruvate [25]. In fact, several experiments aiming at identifying mitochondrial partners of TRXs identified more than 100 putative targets including all enzymes of the TCA cycle and enzymes involved in many other processes [22,23,28]. In addition, one should also consider as putative partners, proteins forming intra- or intermolecular disulfide bridges [29], and proteins subject to other redox post-translational modifications (sulfenylation, nitrosylation, glutathionylation) as recently repertoried for photorespiratory and associated enzymes [30].

In order to understand whether the two mitochondrial TRXs o from *A. thaliana* could be distinguished by their biochemical properties, we have performed a structure–function analysis of both isoforms. We observed that both proteins bind an Fe-S cluster when expressed as recombinant proteins in *Escherichia coli*. The Fe-S cluster ligation in TRXo2 depends on the cysteines found in the conserved WCGPC motif. The physiological relevance of this observation remains unclear. Another observation that may give hints towards a possible connection with the mitochondrial Fe-S cluster assembly machinery is that apo-TRXs o have the capacity to reduce oxidized NFU4 or NFU5, proteins previously isolated as putative TRX partners and known to participate in the maturation of certain mitochondrial Fe-S proteins [1,2,3]. Although solving their respective 3D structures indicated that marked differences in the surface charge distribution exist between both proteins, no difference between TRXo1 and TRXo2 was observed in the capacity to reduce NFU proteins.

## 2. Materials and Methods 

### 2.1. Cloning and Site-Directed Mutagenesis

The sequences coding for the presumed mature form (i.e., devoid of N-terminal targeting sequences) of *A. thaliana* TRXo1 (At2g35010) and TRXo2 (At1g31020) were cloned into the *Nde*I and *Bam*HI restriction sites of both pET12a and pET15b. The cysteines of TRXo2 were individually substituted into serines by site-directed mutagenesis by primer extension using two complementary mutagenic primers [31,32]. In a first round of PCR, two fragments with overlapping ends are generated using TRXo2 forward and mutagenic reverse primers and TRXo2 reverse and mutagenic forward primers, respectively. For the second round of PCR, these fragments were mixed with TRXo2 forward and reverse primers added after 10 PCR cycles to obtain the final product with the desired mutation. The corresponding variants were named TRXo2 C37S and C40S. The sequences coding for the presumed mature forms of AtNFU4 (At3g20970) and AtNFU5 (At1g51390) were cloned into the *Nco*I and *Bam*HI restriction sites of pET3d and the sequence coding for *E. coli* IscS was cloned into the *Nde*I and *Bam*HI restriction sites of pET12a. All primers used in this study are listed in Table S1.

### 2.2. Heterologous Expression in Escherichia coli and Purification of Recombinant Proteins 

For protein production, the *E. coli* BL21 (DE3) strain containing the pSBET plasmid, which allows expression of the transfer ribonucleic acid (tRNA) needed to recognize the AGG and AGA rare codons [33], was co-transformed with recombinant pET3d and pET12a plasmids to produce untagged proteins and with recombinant pET15b plasmids to produce N-terminal His-tagged proteins. The volumes of cultures of *E. coli* transformed cells were progressively increased up to 2.4 L in LB medium at 37 °C supplemented with 50 µg/mL of ampicillin and kanamycin. Protein expression was induced at exponential phase by adding 100 µM isopropyl β-d-thiogalactopyranoside for 4 h at 37 °C. Cultures were then centrifuged for 20 min at 6318 g and the cell pellets were resuspended in about 20 mL of Tris NaCl (30 mM Tris-HCl pH 8.0, 200 mM NaCl) for untagged proteins or TI NaCl (50 mM Tris-HCl pH 8.0, 10 mM imidazole, 300 mM NaCl) for His-tagged proteins and conserved at −20 °C. Cell lysis was performed by sonication (3 × 1 min with intervals of 1 min) and the soluble and insoluble fractions were separated at 4 °C by centrifugation for 30 min at 27,216× *g*.

For His-tagged TRXs o, the soluble fraction was loaded on Ni^2+^ affinity columns (Sigma-Aldrich, St Louis MO, USA). After extensive washing, proteins were eluted by adding 50 mM Tris-HCl pH 8.0, 300 mM NaCl, 250 mM imidazole. The recombinant proteins were concentrated by ultrafiltration under nitrogen pressure and dialyzed (Amicon, YM10 membrane, Merck, Berlington MA, USA) and stored in a 30 mM Tris-HCl pH 8.0 buffer supplemented with 50% glycerol at −20 °C. The purification of His-tagged proteins was performed in aerobic or anaerobic conditions. Anaerobic manipulations were performed in a Jacomex glovebox under a nitrogen atmosphere with oxygen levels below 1 ppm. 

For untagged proteins, the soluble fraction was first precipitated by ammonium sulfate from 0 to 40% and then to 80% of the saturation. Both TRXo and NFU proteins precipitated mostly between 40 and 80% of ammonium sulfate saturation and *E. coli* IscS between 0 and 40%. The fractions of interest were subjected to size exclusion chromatography (ACA44 for TRXo and NFU proteins, ACA34 for IscS) equilibrated with a Tris NaCl buffer. After dialysis against a 30 mM Tris-HCl pH 8.0 buffer and concentration, the interesting fractions were loaded to a DEAE (diethylaminoethyl) sepharose column equilibrated with the same buffer. The recombinant TRXo proteins that passed through the DEAE column, were concentrated by ultrafiltration under nitrogen pressure (Amicon cells, YM10 membrane), and were stored in the same buffer in the presence of 50% glycerol at −20 °C. On the contrary, NFU and IscS proteins were retained and eluted using a linear 0–0.4 M NaCl gradient. The purest fractions as judged by sodium dodecyl sulfate polyacrylamide gel electrophoresis (SDS-PAGE) analysis were pooled and dialyzed against 30 mM Tris-HCl pH 8.0 buffer as described above.

Protein concentrations were determined spectrophotometrically using a molecular extinction coefficient at 280 nm of 11,585 M^−1^ cm^−1^ for TRXo1, TRXo2, and NFU5; of 11,460 M^−1^ cm^−1^ for TRXo2 cysteine variants; of 13,075 M^−1^ cm^−1^ for NFU4; and of 41,495 M^−1^ cm^−1^ for IscS, as determined from the molar extinction coefficients of individual tyrosines, tryptophans, and cystines (1490, 5500, and 125 M^−1^ cm^−1^, respectively) [34].

The other recombinant proteins used in this work e.g., NTRB and GRXS15 from *A. thaliana* have been purified as described previously [35,36].

### 2.3. Preparation of Apo-TRXs o and IscS-Mediated *In Vitro* Fe-S Cluster Reconstitution of TRXs o

Under strictly anaerobic conditions in a glove box, 200 µM untagged apo-TRXo isoforms (proteins obtained after aerobic purification) were incubated with 100-fold excess of dithiothreitol (DTT) in Tris NaCl buffer for 1 h. The Fe-S cluster reconstitution was then initiated by the addition of catalytic amount of EcIscS (0.05 fold, 10 µM), five-fold excess of ferrous ammonium sulfate citrate (1 mM) and finally five-fold excess of L-cysteine (1 mM). After 30 min of incubation, the Fe-S cluster-loaded TRXo proteins were desalted on a G25 column equilibrated with Tris NaCl buffer.

### 2.4. Determination of the Oligomerization State

The oligomerization state of TRXo isoforms was analyzed by size-exclusion chromatography. Samples containing 100 to 300 µg of protein were loaded onto Superdex S200 10/300 columns equilibrated with 30 mM Tris-HCl pH 8.0, 200 mM NaCl and connected to an Äkta purifier system (GE Healthcare). The flow rate was fixed at 0.5 mL min^−1^, and detection was recorded at 280 and 420 nm. The column was calibrated using a molecular weight standard from Sigma. Protein names, molecular weights and elution volumes are as follows: thyroglobulin 669 kDa, 8.47 mL; apoferritin 443 kDa, 10.51 mL; β-amylase 200 kDa, 11.92 mL; bovine serum albumin 66 kDa, 13.56 mL; carbonic anhydrase 29 kDa, 15.54 mL; cytochrome c 12.4 kDa, 16.82 mL and aprotinin 6.5 kDa, 18.16 mL.

### 2.5. GRX- and TRX-Mediated Reduction of NFUs

Around 3 mg of NFU proteins were reduced using 10 mM DTT in 200 µL of 30 mM Tris-HCl pH 8.0 buffer for 1 h at 25 °C. The reduced proteins were then desalted on a G25 column pre-equilibrated with 30 mM Tris-HCl pH 8.0 buffer. The redox state of untreated and reduced NFUs was determined by electrospray ionization mass spectrometry analysis as described previously [37], and by a cysteine alkylation assay, which also served for assessing reduction by the TRX and GRX reducing systems. The TRX system reconstituted in vitro comprised 200 µM NADPH, 100 nM NTRB, and 1 or 10 µM untagged TRXo1 or o2. The GSH/GRX system was composed of 200 µM NADPH, 0.1 units GR from bakers’yeast (Sigma-Aldrich, St Louis MO, USA), 1 mM GSH and 1 or 10 µM GRXS15. Each reducing system was incubated at 25 °C for 15 min prior addition of 10 µM of oxidized NFU4/5 in 50 µL of 30 mM Tris-HCl pH 8.0 buffer. The reaction was stopped after 15 min by the addition of one volume of 20% TCA. Protein free thiol groups have been alkylated with methoxyl-PEG (mPEG)-maleimide of 2 kDa as described previously [38], before separating the protein mixtures on non-reducing 15% SDS-PAGE.

### 2.6. Crystallization, Diffraction Data Collection, Processing, Structure Solution, and Refinement

Crystallization at 4 °C was performed by a microbatch-under-oil method, in which 1 µL of the protein solutions were mixed with an equal volume of the precipitant solution containing 100 mM HEPES pH 7.5 and 20% PEG 8000 for TRXo1 and 100 mM citrate buffer pH 5.6, 30% PEG 4000 and 100 mM ammonium sulfate for TRXo2. Solutions of TRXo1 and TRXo2 had concentrations of 20 mg mL^−1^ and 13 mg mL^−1^, respectively. Suitable crystals were soaked briefly in crystallization solution supplemented with 20% glycerol and then flash-frozen immediately in a nitrogen stream before data collection. X-ray diffraction experiments were carried out at 100 K on the French Beamline for Investigation of Proteins–Beam Magnet 30A (FIP-BM30A) at the European Synchrotron Radiation Facility, Grenoble, France. The data sets at 1.80 and 1.50 Å for TRXo1 and TRXo2, respectively, were indexed and processed using XDS [39], and scaled and merged with Aimless from the CCP4 program package [40]. The initial protein model of TRXo2 was built in ARP/wARP [41]. The crystal structure of TRXo1 was solved by molecular replacement with the CCP4 suite program (MOLREP) [42], using the structure of TRXo2 as a search model. Structures were refined (Table 1) with phenix.refine implemented in the PHENIX package [43], with visual inspection and manual correction in Coot [44]. Structures of TRXo1 and TRXo2 were refined to a crystallographic R_work_ and R_free_ of 0.22/0.24 and 0.17/0.18, respectively, by using standard protocols, in which 5% of the reflections were used to continuously monitor the R_free_. The refined structures maintained excellent geometry and showed no outlier in Ramachandran plots. The validation of the crystal structures was performed with MolProbity [45].

## 3. Results

### 3.1. Arabidopsis thaliana TRXo Isoforms Exist in Two Forms upon Expression in E. coli

**Figure 1:**
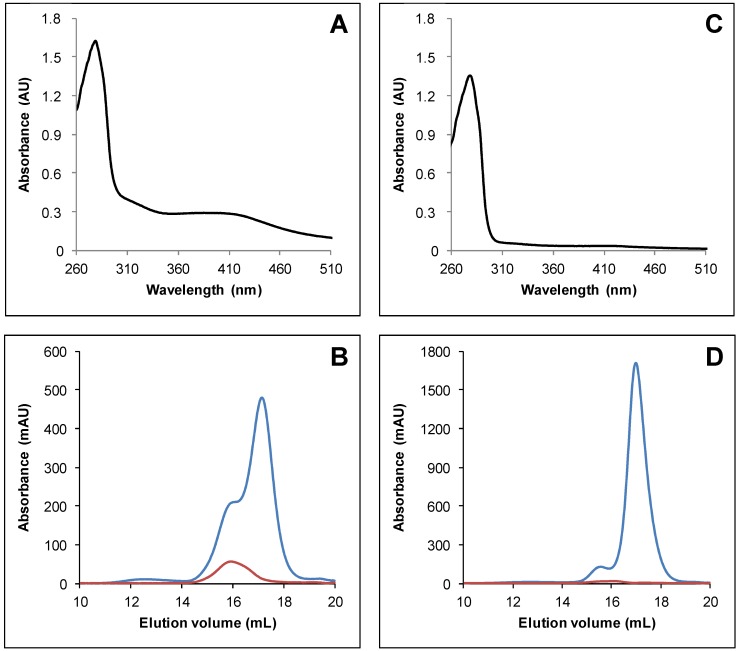
Arabidopsis TRXo (thioredoxin-*o*) isoforms incorporate an Fe-S cluster after heterologous expression in *E. coli*. UV–visible absorption spectra of His-tagged recombinant TRXo2 (**A**) and TRXo1 (**C**). Spectra were recorded after an anaerobic purification in 30 mM Tris-HCl pH 8.0. Analytical gel filtration of His-tagged recombinant TRXo2 (**B**) and TRXo1 (**D**), purified in anaerobic conditions, was performed by loading 100 to 300 µg of protein onto a Superdex S200 10/300 column. The presence of the polypeptide and of the Fe-S cluster have been detected by the absorbance at 280 nm (blue line) and 420 nm (red line), respectively.

To characterize the structure–function relationship of TRXo1 and TRXo2, the mature forms of these proteins were expressed in *E. coli* as untagged and His-tagged recombinant proteins by removing respectively 82 and 47 residues at the N-terminus constituting their putative mitochondrial targeting sequence. As observed for several related proteins of the GRX family [37,46], lysed bacterial cells exhibited a slight but visible brownish color typical of the presence of an Fe-S cluster. After purification in aerobic conditions, His-tagged recombinant proteins purified in one step displayed a residual brownish color unlike untagged versions (data not shown). The reason was obviously associated to the oxygen lability of the cluster and to the length of the purification procedure. These observations prompted us to purify both His-tagged TRXs in anaerobic conditions to avoid chromophore degradation. TRXo2 exhibited an UV–vis absorption spectrum comprising a broad shoulder centered at around 400 nm and a band at around 300 nm that prolonged the polypeptide absorption band at 280 nm (Figure 1A). This spectrum is usually typical of the presence of a [4Fe-4S] cluster [47,48]. Analytical gel filtration analyses demonstrated that TRXo2 (theoretical molecular mass of ca 14.5 kDa) separated into two peaks, a major one with an estimated molecular mass of 12.9 kDa and a minor one of 27.7 kDa suggesting that the purified protein solution contained both monomeric and dimeric forms (Figure 1B). Furthermore, the Fe-S cluster, detected by the absorbance at 420 nm, was only associated with the dimeric form (Figure 1B). Using a similar protocol, TRXo1 displayed a nearly identical UV–vis absorption spectrum, albeit absorption bands in the visible region were much less intense (Figure 1C). Moreover, TRXo1 (theoretical mass of ca 14.6 kDa) also separated into two peaks when analyzed by analytical gel filtration, a major one with an estimated molecular mass of 12.9 kDa and a minor one of 24.8 kDa which corresponded to a apomonomer and a holodimer form, respectively (Figure 1D). Altogether, these results indicate that both TRXo isoforms incorporate an Fe-S cluster after heterologous expression in *E. coli*. Despite these similarities, it seems that the Fe-S cluster in TRXo1 is either less-well maturated by the *E. coli* Fe-S cluster assembly machineries or more oxygen-labile as cell lysis is performed in the presence of oxygen.

### 3.2. IscS-Mediated *In Vitro* Fe-S Cluster Reconstitution in Arabidopsis TRXo Isoforms

**Figure 2:**
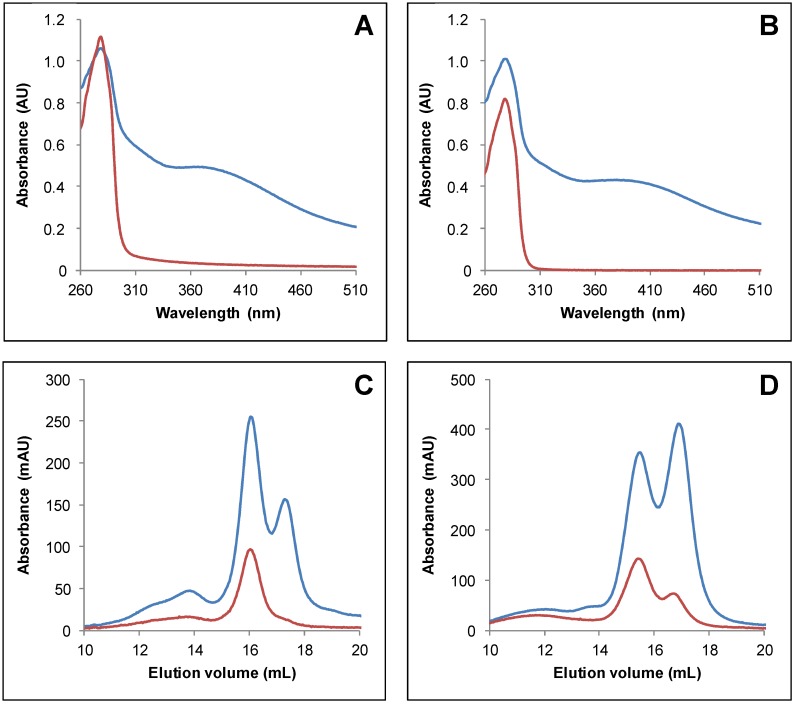
IscS-mediated in vitro Fe-S cluster reconstitution of Arabidopsis TRXo isoforms. UV–visible absorption spectra of TRXo2 (**A**) and TRXo1 (**C**) before (red line) and after (blue line) an anaerobic reconstitution performed in the presence of IscS in Tris NaCl buffer. Analytical gel filtration of reconstituted TRXo2 (**B**) and TRXo1 (**D**) was performed by loading 100 to 300 µg of protein (including 10 µM of EcIscS) onto a Superdex S200 10/300 column. The presence of the polypeptide and of the Fe-S cluster have been detected by the absorbance at 280 nm (blue line) and 420 nm (red line), respectively.

As purified recombinant Arabidopsis TRXo isoforms existed predominantly under apoforms, we sought to prepare samples by performing in vitro enzymatic Fe-S cluster reconstitution experiments in the presence of the *E. coli* cysteine desulfurase IscS. Untagged apo-TRXo1 and TRXo2 (Figure S1) were incubated anaerobically with an excess of L-cysteine and ferrous ammonium sulfate, and a catalytic amount of IscS for 1 h. Both reconstituted TRXo1 and TRXo2 exhibited an UV–visible absorption spectrum (Figure 2A,B) similar to the one observed for as-purified proteins but with more intense absorption bands around 300 and 400 nm relative to the one at 280 nm. Analytical gel filtration analyses demonstrated that both TRXo1 and TRXo2 still separated into two peaks, one with an estimated molecular mass of 25 kDa and another one of 12.9 kDa (Figure 2C,D). The smaller peak around 14 mL corresponds to IscS protein as deduced from the comparison with the analytical gel filtration performed with an IscS concentration similar to the one used for reconstitution (Figure S2). Although the peak containing the dimeric TRX holoforms was more prominent, monomeric forms were still present in the reconstituted samples. The presence of an absorbance at 420 nm associated with the monomeric TRXo1 may be due to the fact that thiol groups of DTT present in the reconstitution mixture served as ligands. These results indicated that IscS-mediated in vitro reconstitution increased the content of Fe-S clusters in both TRXo isoforms but we could not find conditions to get fully-repleted Fe-S cluster-loaded forms that would allow an accurate analytical titration of Fe and labile sulfide contents. A rough evaluation of the Fe-S cluster content in TRXs o was performed from the absorption band at 410 nm by comparison with data obtained with a fully replete ferredoxin-thioredoxin reductase (FTR) an enzyme, which also contains a [4Fe-4S] center. Indeed, the UV–visible absorption spectra of FTRs purified from different organisms consistently exhibited an A_410_/A_280_ ratio of 0.42 and molar extinction coefficient values at 410 nm of 17,400 and 15,000 M^−1^ cm^−1^ were determined for spinach and corn FTRs [49]. From the comparable A_410_/A_280_ ratio in the UV–visible absorption spectra of TRXs o and FTR and an averaged molar extinction coefficient value at 410 nm of 16,000 M^−1^ cm^−1^ as determined for FTRs, we could deduce that there is ca 30 µM of Fe-S cluster in each TRX o sample formed by a mixture of monomer and dimer. On the other hand, using the theoretical molar extinction coefficients at 280 nm, we estimated that TRX o samples contained approximately 85–90 μM TRXo monomers (Figure 2A,B). Hence, considering that the Fe-S cluster is bridged into TRX o dimers, we concluded that about two-thirds of the proteins are replete with an Fe-S cluster.

### 3.3. Both Active Site Cysteines of TRXo2 Are Required for Fe-S Cluster Incorporation

**Figure 3:**
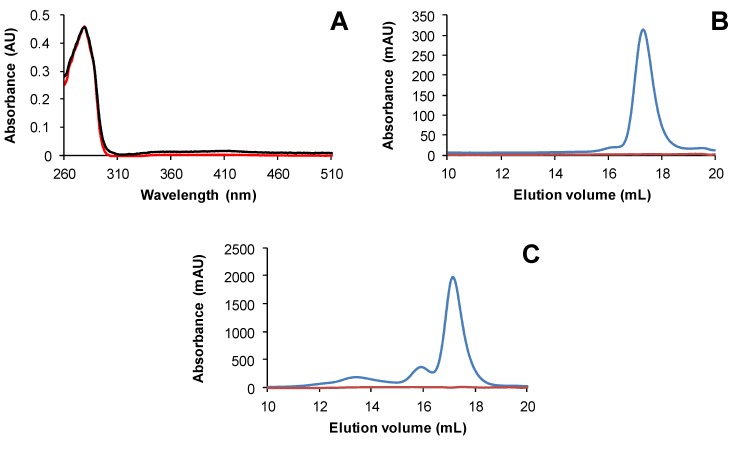
Monocysteinic variants of Arabidopsis TRXo2 isoform are mostly apo-monomers. UV–visible absorption spectra of His-tagged recombinant TRXo2 C37S (red line) and C40S (black line) (**A**) recorded after an anaerobic purification in 30 mM Tris-HCl pH 8.0. Analytical gel filtration of His-tagged recombinant TRXo2 C37S (**B**) and C40S (**C**) purified in anaerobic conditions, was performed by loading 100 to 300 µg of protein onto a Superdex S200 10/300 column. The presence of the polypeptide and of the Fe-S cluster have been detected by the absorbance at 280 nm (blue line) and 420 nm (red line), respectively.

The existence of Fe-S cluster-bridged TRX isoforms were previously observed for the atypical TRX isoform (IsTRP) from the human pathogen *Echinococcus granulosus* [50], and *E. coli* TrxA variants with CACC and CACA active site motifs [51]. The cysteines of the active site signature of IsTRP proved to be essential for cluster binding. Among potential Fe-S cluster ligands, both TRXs o have in common the two active site cysteines but no extra cysteine. To investigate the role of these cysteines for Fe-S cluster ligation, His-tagged C37S and C40S variants of TRXo2 were purified under anaerobiosis. Unlike TRXo2, no coloration was visible on as-purified TRXo2 C37S and C40S variants and accordingly no other absorption band than the one centered at 280 nm was detected on their UV–vis absorption spectra (Figure 3A). Analytical gel filtration experiments revealed that the TRXo2 C37S variant separated as a single peak with an apparent molecular mass of 12.2 kDa likely corresponding to an apomonomer, whereas a small additional peak (15.8 mL, 24.9 kDa) was observed for the TRXo2 C40S variant, which likely contained a covalent dimeric form with Cys^37^ of two monomers forming an intermolecular disulfide (Figure 3B,C). Altogether these results suggest that the substitution of Cys^37^ and Cys^40^ hampered Fe-S cluster incorporation in TRXo2 underlying that both cysteines are required for Fe-S cluster binding.

### 3.4. TRXo1 and TRXo2 Possess the Structural Properties of TRX Family Members 

To definitely confirm the identity of the Fe-S cluster ligands and decipher the other structural features of TRXo isoforms for which there is no known 3D structure solved so far, we tried to crystallize under nitrogen atmosphere both TRXo1 and TRXo2 holoforms. Crystals have been obtained for both proteins but it turned out that they were not colored and contained only apoforms. TRXo1 crystallized in the orthorhombic space group *P*2_1_2_1_2_1_ whereas TRXo2 crystallized in the hexagonal system and belonged to space group *P*6_5_. Well-diffracting crystals of TRXo1 and TRXo2 were obtained with one molecule per asymmetric unit (a.s.u) with Matthews’s coefficient (Vm) values of 2.3 Å^3^ Da^−l^ and 2.0 Å^3^ Da^−l^ corresponding to solvent contents of 46% and 40%, respectively. Their structures were determined by molecular replacement at high resolution of 1.80 and 1.50 Å, respectively. In both protein structures, almost all residues were well defined in the electron density map, except the first two residues (Met^1^ and Glu^2^) of TRXo1, which were disordered (amino acid numbering refers to the mature protein without its mitochondrial presequence). 

**Figure 4:**
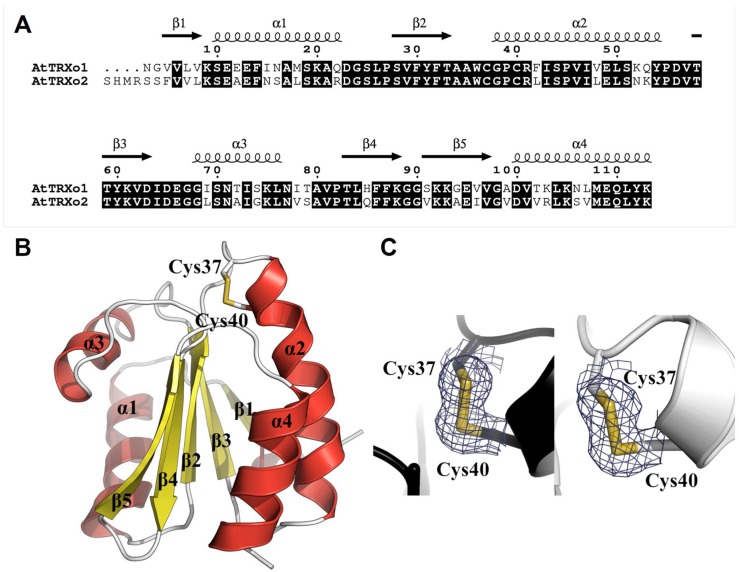
Three-dimensional structure and sequence conservation of oxidized Arabidopsis TRXo isoforms. (**A**) Structure-based sequence alignment of Arabidopsis TRXo isoforms. Conserved residues are highlighted in black. (**B**) Three-dimensional structure of AtTRXo2 at 1.50 Å resolution. The X-ray structure of AtTRXo2 is shown as a ribbon representation with helices in red and strands in yellow. In addition, the side chains of Cys^37^ and Cys^40^ residues are shown as sticks. (**C**) Electron density around the Cys^37^-Cys^40^ disulfide bond. The maps shown are σA-weighted 2*mF_o_*-*DF_c_* maps contoured at 1.2σ (0.07 and 0.38 e/Å^3^ for AtTRXo1 and AtTRXo2, respectively). AtTRXo1 and AtTRXo2 are colored in white and black, respectively.

Both proteins adopt the overall TRX fold (βαβαβαββα) with all regular secondary structure elements preserved and consisting of a central core with a twisted five-stranded β-sheet in which β4 is antiparallel to the others (Figure 4A,B). This β-sheet is capped on one side by α1 and α3 helices and on the other side by α2 and α4 helices (Figure 4B). All secondary structures of these proteins (TRXo1 numbering) are β1 (Val^5^-Val^8^), α1 (Ser^10^-Gln^22^), β2 (Ser^28^-Thr^33^), α2 (Gly^38^-Tyr^54^), β3 (Thr^58^-Asp^63^), α3 (Gly^68^-Leu^76^), β4 (Thr^83^-Lys^88^), β5 (Ser^91^-Val^97^), and α4 (Asp^100^-Lys^113^). TRXs generally contain a buried Asp^31^ near Cys^40^ residue, which is replaced by Tyr^31^ in Arabidopsis TRXo isoforms as in *Anabaena* Trx2 [52]. The position of the sidechain hydroxyl of Tyr^31^ corresponds very closely to a water-mediated hydrogen bonding between the classical Asp and the Cys^40^ Sγ. In TRXo1 and TRXo2 X-ray structures, the distance between the sidechain hydroxyl of the Tyr^31^ and the Cys^40^ Sγ atom are 3.76 and 5.51 Å, respectively, too far away for an interaction.

The Cys^37^ and Cys^40^ residues are found at the end of the α2-helix, the Cys^40^ residue being buried compared to Cys^37^. Close examination of the resultant Fo-Fc electron density maps revealed a negative density between Cys^37^ and Cys^40^ in TRXo2, and additional positive density in the opposite sides of Sγ atoms. Refinements, using PHENIX and Coot in an iterative manner, indicated that this effect is an artifact of data collection (radiation damage), due to partial reduction of the disulfide bond by the X-ray beam during data collection. The distances between Cys^37^ and Cys^40^ Sγ atoms are 2.03 and 2.05 Å in TRXo1 and TRXo2, respectively. They represent the typical length of disulfide bonds, suggesting that both TRXo isoforms were crystallized in an oxidized form. Hence, we modeled all the cysteine pairs as forming intramolecular disulfide bonds (Figure 4C). 

### 3.5. TRXo1 and TRXo2 Structures Are Not Strictly Superimposable 

**Figure 5:**
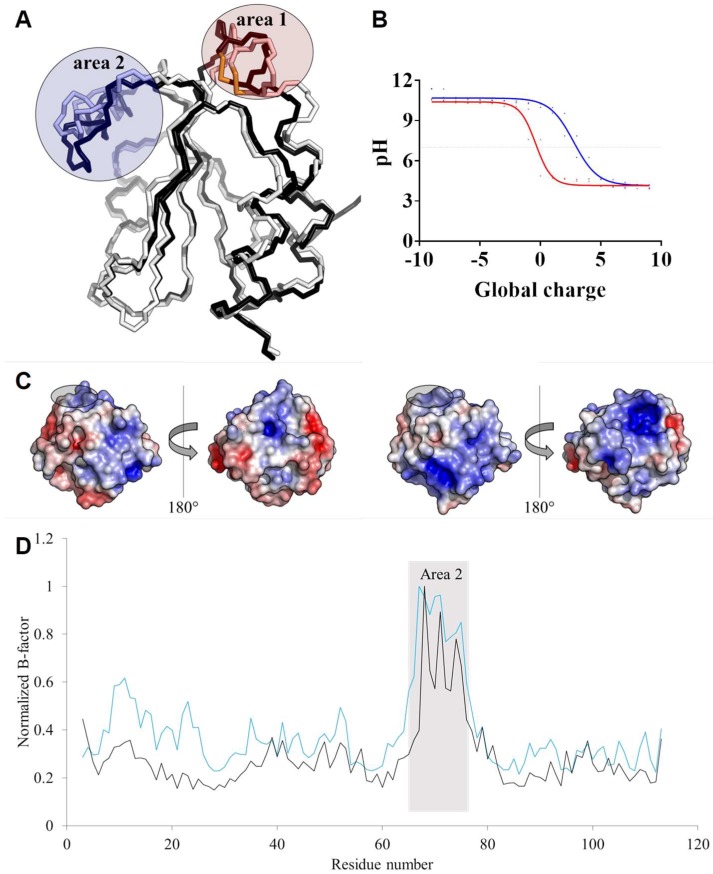
Structural features *vs.* charge and crystallographic B-factor distribution. (**A**) Structure superposition of the backbone trace of Arabidopsis TRXo isoforms. AtTRXo1 and AtTRXo2 are colored in white and black, respectively. The two divergent areas are circled in red and blue, respectively. (**B**) Global protein charge of AtTRXo1 (red) and AtTRXo2 (blue) as a function of pH as indicated by the PDB2PQR Server [54]. The dot line corresponds to pH 7.0. (**C**) Electrostatic potential mapped onto AtTRXo1 (left) and AtTRXo2 (right) structures at pH 8.1. The WCGPC signature residues are circled in black. (**D**) Flexibility of AtTRXo1 (black) and AtTRXo2 (blue) related to the crystallographic B-factor.

TRXo1 and TRXo2 share high levels of sequence identity and similarity (74% and 97%, respectively) (Figure 4A) and their crystal structures are quite similar and overlay well (RMSD of 0.9 Å) (Figure 5A). Nevertheless, a fine comparison of both structures revealed two regions presenting significant structural differences. The first area corresponds to the active site loop, which protrudes on one side of the molecule to the N-terminus of α2-helix (Figure 5A). The region containing Trp^36^-Phe^42^ residues including the WCGPC signature in TRXo1 has a different conformation to that in TRXo2 and is shifted by approximately 1.9 Å. Trp^36^ and Arg^41^ residues are involved in contacts with symmetric molecules through hydrogen bond stabilization. In both proteins, the carbonyl oxygen of Trp^36^ forms a hydrogen bond with the Nζ atom of Lys^105^ (2.81 Å in TRXo1 and 3.15 Å in TRXo2). The NH1 guanidinium nitrogen of Arg^41^ is hydrogen-bonded to the carbonyl oxygen of Ser^74^ (2.85 Å) in TRXo1 and to the carbonyl oxygen of Ala^12^ (3.50 Å) in TRXo2. This regional difference around the active site is near crystal contacts and may be attributable to different crystal packing interactions. On another note, the fully refined TRXo1 structure contains 130 water molecules whereas the TRXo2 structure includes 92 water molecules, of which 51 have equivalent positions in TRXo1. Despite the high degree of conservation of both primary and tertiary structures between TRXo1 and TRXo2, only 39% and 55% of the water molecule positions are conserved indicating probable differences in the charge state distribution. Even though TRXo1 was crystallized at pH 7.5 whereas TRXo2 was crystallized at pH 5.6, which may explain differences in the solvation shell, TRXo2 would be more positively charged than TRXo1 in a pH range between 5 and 10 (Figure 5B). With a pH value of 8.1 ± 0.2 for the mitochondrial matrix of *A. thaliana* [53], the global charge of TRXo1 and TRXo2 in vivo should be quite different with values of −1 and +2, respectively. The differences in the distribution of the electrostatic potential on the TRXo1 and TRXo2 surfaces at pH 8.1 are however not located in the area surrounding the active site (Figure 5C). The second divergent area involves Gly^68^-Thr^79^ residues (Figure 5A) with an RMSD of 2.6 Å. This area surrounds the α3-helix upstream of the conserved *cis*-Pro^82^ residue, which is located at the N-terminus of β4-strand. The increase of the crystallographic B-factor along the amino acid chain for TRXo1 (65.33 Å²) and TRXo2 (68.91 Å²) (Figure 5D), indicates that this part is flexible. Altogether, this may hint to a capacity of TRXo isoforms to interact with different partner proteins.

### 3.6. Oxidized NFU4 and NFU5 Are Reduced by TRXo Isoforms but Not by GRXS15

With the aim of confirming novel TRXo partners, we examined the 101 putative TRXo1 partners identified in a previous proteomic analysis performed by affinity chromatography using a TRXo1 C40S variant and mitochondrial protein extracts [22]. The DTT-dependent elution of these proteins suggests that these TRX partners were trapped because they existed under oxidized forms, which led to the formation of mixed-disulfide intermediates with the TRXo1 variant. Among these proteins, we focused our attention on two proteins, NFU4 and NFU5, involved in the late steps of the maturation of Fe-S proteins and acting as Fe-S cluster transfer proteins. These proteins possess a CxxC motif, the cysteines of which being responsible of the transient ligation of the Fe-S cluster. Thus, they have to be reduced to receive the Fe-S cluster from other maturation factors.

The mature forms of both NFU proteins have been expressed in *E. coli* and purified aerobically. As expected, signs for the presence of an Fe-S cluster were initially visible, but both NFU4 and NFU5 were found as apoproteins at the end of the purification. They have three cysteines including those of the CxxC motif. To determine their oxidation state, mass spectrometry analyses were performed with NFU4 and NFU5 either untreated or reduced by a DTT excess and dialyzed (Figures S3 and S4). A single species was obtained for both untreated proteins but it presented a mass decrease of ca 131 Da compared to their theoretical molecular masses (Table 2). This difference corresponds undoubtedly to the cleavage of the first methionine as expected from the presence of an alanine at the second position. An increase of around 2 Da in the molecular masses of reduced proteins likely corresponded to the gain of two protons indicating the existence of an intramolecular disulfide bond in as-purified proteins (Table 2).

**Figure 6:**
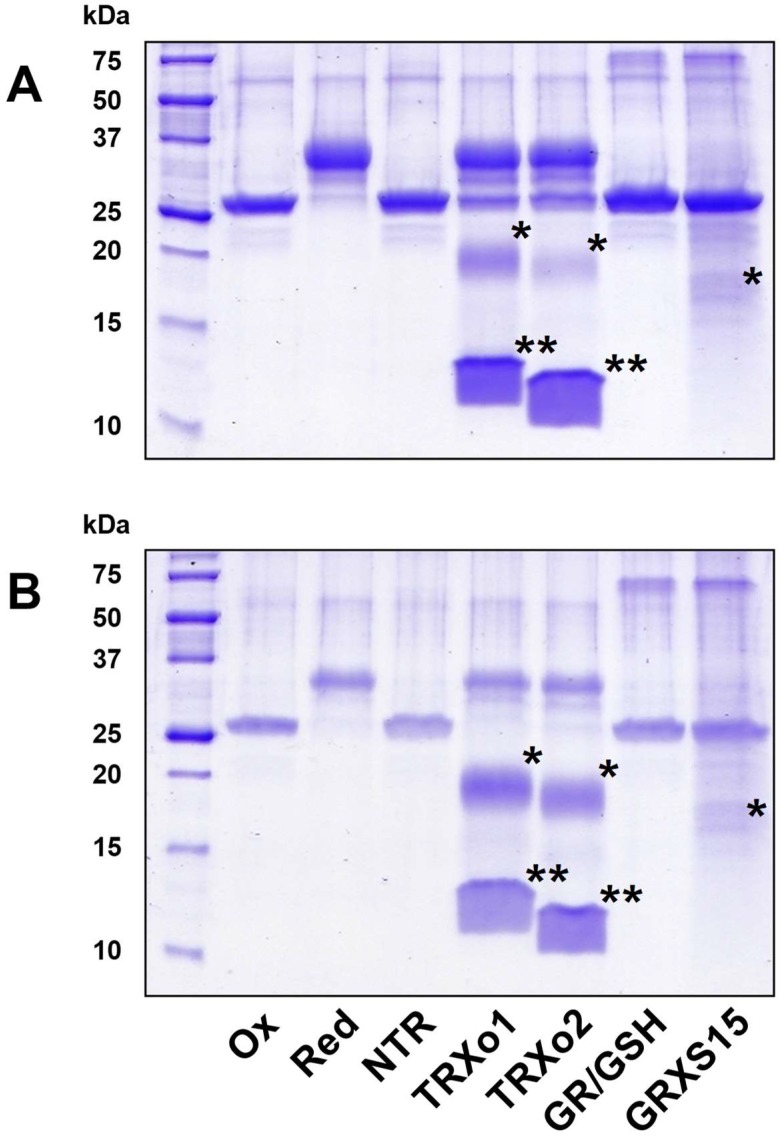
The disulfide bridge of mitochondrial NFUs (nitrogen-fixation-subunit-Us) is reduced by TRXs o but not GRXS15. The reduction of as-purified, oxidized forms of NFU4 (**A**) or NFU5 (**B**) was assessed after a 15 min incubation in the presence of the following reducing systems: NTR: NADPH + NTR; TRXo1: NADPH + NTR + TRXo1; TRXo2: NADPH + NTR + TRXo2; GR/GSH: NADPH + GR + GSH ; GRXS15: NADPH + GR + GSH + GRXS15. After alkylation with 2 kDa mPEG maleimide, proteins were separated on non-reducing SDS-PAGE (sodium dodecyl sulfate polyacrylamide gel electrophoresis). Reduced (Red) and oxidized (Ox) proteins served as controls. The stars indicate the alkylated (*) and non-alkylated (**) forms of the oxidoreductases in the respective regeneration systems when visible.

To examine the ability of the various reducing systems found in mitochondria to reduce oxidized NFU proteins, untreated NFU4 and NFU5 were incubated with TRX or GSH/GRX reducing systems. Subsequent alkylation of thiol groups with 2 kDa mPEG maleimide and separation on non-reducing SDS-PAGE allowed visualizing the redox state of the proteins (Figure 6). After 15 min reaction, the NADPH/GR/GSH system was clearly unable to reduce oxidized NFU proteins and adding the sole mitochondrial GRX, GRXS15, did not improve the reduction. On the contrary, in the presence of NADPH and NTR, both TRXo isoforms reduced completely oxidized NFU proteins when added at equimolar concentrations (Figure 6). Adding more catalytic amounts of TRXs o by decreasing the relative concentrations of TRXs o vs. NFUs to 1:10 still allowed an efficient reduction of both NFUs, although this was not complete (Figure S5). In the presence of a NADPH/NTR regeneration system, GRXS15 was still not able to reduce oxidized NFUs (Figure S5). These in vitro data indicated that the reduction of oxidized mitochondrial NFU isoforms, would this oxidation occur under specific physiological conditions, would depend on the TRX system but not on GSH/GRX or NTR/GRX systems. No difference between both TRXs o was visible using this assay.

## 4. Discussion and Conclusions

Compared to plastidial TRX isoforms, the molecular and physiological roles of mitochondrial TRXo isoforms are uncertain although numerous mitochondrial proteins are known or assumed to undergo reversible redox post-translational modifications and a hundred of putative TRXo1 partners have been identified [22]. In *A. thaliana*, TRXo1 is the major TRXo isoform but *A. thaliana trxo1* and *trxo2* single and double mutants have no pronounced phenotype [25,26]. Because previous studies did not provide information about the redundancy or specificity of these isoforms, we have decided to investigate the biochemical and structural properties of both proteins. Given that TRXo1 and TRXo2 have been previously purified as recombinant proteins [22], and possess the regular WCGPC signature found in many TRXs, it was completely unexpected that both recombinant proteins formed homodimers bridging a [4Fe-4S] cluster using cysteines of the active site signature either upon expression in *E. coli* or upon in vitro Fe-S cluster reconstitution experiments. To our knowledge, this is the first report that a non-modified TRX incorporates a [4Fe-4S] cluster. Up to now, only a few reports pointed to the capacity of proteins of the TRX superfamily to bind an Fe-S cluster but never a [4Fe-4S] cluster in regular TRXs. A thioredoxin-related protein from the human pathogen *Echinococcus granulosus*, IsTRP, was shown to bind a [2Fe-2S] cluster using cysteines present in an atypical active site signature (NCFAC) [50]. Two atypical PDI-A isoforms from poplar and Arabidopsis, formed by a single domain and possessing a WCKHC signature, are able to bind a [2Fe-2S] cluster into a homodimer using the cysteines present in this active site signature [55,56]. Several glutaredoxins can bind a [2Fe-2S] cluster into homodimers using the first cysteine of the CxxC/S signature and the thiol group of two glutathione molecules [37,46,57,58,59] Interestingly, *S. cerevisiae* GRX5 also binds a [4Fe-4S] cluster in the absence of glutathione, using a cysteine present in the C-terminal part [48].

Besides these naturally-existing isoforms, several variants of *E. coli* or human TRX1 were shown to bind various types of Fe-S cluster. By introducing two mutations, W28C and I75C, a mononuclear [FeS4] cluster can be incorporated in *E. coli* Trx1 both in vitro and in vivo using also the two active site cysteines, Cys^32^ and Cys^35^ [60]. By searching proteins that could restore disulfide bond formation when exported to the periplasm of strains lacking the entire periplasmic oxidative pathway, two *E. coli* Trx1 variants with CACC and CACA active site motifs were shown to form Fe-S cluster-bridged homodimers with the cysteines forming the CxC motif being essential for cluster ligation [51]. The structure of an apo Trx1 CACA variant, where the cysteines thought to serve as ligands of the Fe-S cluster are engaged in two intermolecular disulfide bonds, has been solved [61]. It seems that the exposure of the second active site cysteine, generally buried in TRXs, caused by the unraveling of α2 helix may be sufficient to enable Fe-S cluster binding. Whether a similar change occurs in the structures of TRXo is unknown. Last but not least, a [4Fe-4S] cluster can be assembled into the hydrophobic core of a monomeric *E. coli* Trx1 [62]. The polypeptide contained several substitutions for introducing the Fe-S cluster binding residues (L24C, L42C, V55C, and L99C) and removing the two catalytic cysteines. The incorporation was achieved into a fully reduced, partially unfolded protein (2 M urea treatment) from a synthetic, preformed tetranuclear Fe-S cluster. In other words, it required drastic changes and conditions. Very interestingly, in human TRX1, which possesses a regular WCGPC signature, the simple mutation of the *cis*-Pro (P75S/T/A) found in the active site of most TRX superfamily members is sufficient to allow incorporation of a [2Fe-2S] cluster into homodimers [63]. Among the five cysteines present in human TRX1, it seems that Cys^32^ and the specific Cys^73^ are important for iron atom binding. Introducing a glutaredoxin active site (CSYC) into human TRX1, without substituting the *cis*-Pro, allowed Fe-S cluster incorporation. Hence, all these mutagenesis studies performed using *E. coli* Trx1 and human TRX1 suggested that the combination of a WCGPC active site motif and a *cis*-Pro prevented the active site cysteines from binding an Fe-S cluster. However, both Arabidopsis TRXs o have these two features and the capacity to bind an Fe-S cluster indicating that other factors come into play. In order to understand the structural factors that favor Fe-S cluster incorporation into TRXs o, we sought to solve their structures but could only get information on the apoforms so far. 

**Figure 7:**
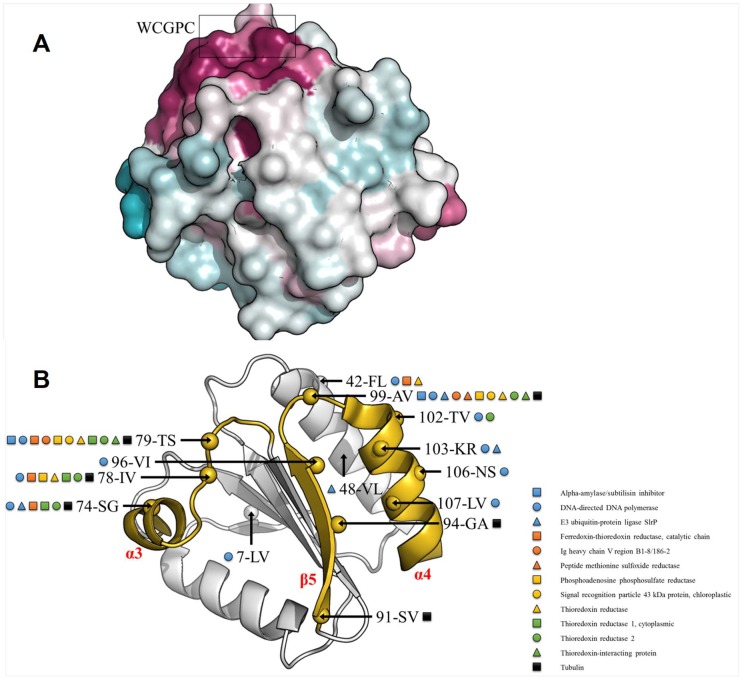
Thioredoxin interfacing residues. (**A**) Residue conservation among 500 thioredoxin orthologs of AtTRXo1 and AtTRXo2 using the ConSurf server [67] with the UniRef90 database (www.uniprot.org/uniref/). Residues are colored in white to purple, for least to most conserved residues. The conserved WCGPC motif is circled in black. (**B**) AtTRXo1 and AtTRXo2 sequences were blasted against the PDB to find protein–protein interactions involving thioredoxin homologs. 44 complexes were found using an E-Value Cutoff of 0.001. AtTRXo1 and AtTRXo2 non-conserved residues potentially involved in protein–protein interactions were mapped onto the X-ray structure of AtTRXo1. In each case the residue position, and the amino acids in one letter code for AtTRXo1 and AtTRXo2 are shown (ex: 78-IV; position 78, isoleucine and valine found in AtTRXo1 and AtTRXo2, respectively). The area comprising α3-helix, the loop between α3 and β4, β5-strand, and α4-helix is colored in yellow.

Although both TRXs o display globally similar structures, interesting observations have been made when the 28 residues varying between Arabidopsis TRXo isoforms were mapped onto AtTRXo1 X-ray structure. These non-conserved residues are solvent exposed and outside the highly conserved area (Figure 7A) comprising the WCGPC active site motif and residues that surround it. However, half of these non-conserved residues is potentially involved in protein–protein interactions (Figure 7B) as determined by a comparison with already characterized complexes involving thioredoxin homologs. These 14 non-conserved residues are mainly located on the same face of the protein (comprising α3-helix, the loop between α3 and β4, β5-strand, and α4-helix) with marked differences in the surface charge distribution (Figure 5C, left panels). The fact that (i) this region is involved in the interaction between TRXs and other proteins [64], (ii) that at mitochondrial pH, TRXo1 (calculated pI of 6.22) would be negatively charged whereas TRXo2 (calculated pI of 10.12) would be positively charged and (iii) that interactions between TRX and their partners are mostly electrostatic could point to a distinct ability of both TRXs o to interact with their partners. However, whether TRXo2 could have specific partners is unknown as only TRXo1 was used to isolate putative partners so far and both isoforms have not been systematically tested with the same proteins. Using the two identified mitochondrial Fe-S cluster maturation factors, NFU4 and NFU5, we could not demonstrate a difference between TRXo1 and TRXo2, both being able to efficiently reduce the intramolecular disulfide formed on NFUs, unlike GRXS15. However, regardless whether a specificity exists, the fact that several ISC factors (NFS1, ISCA4, ISU1) involved in the maturation of Fe-S proteins in mitochondria were identified as putative partners of TRXo1 [22], raises the question of whether TRXo isoforms are involved in the reduction of these proteins in specific conditions where they can become oxidized. Indeed, most proteins involved in the maturation of Fe-S proteins, including NFU4 and NFU5, possess critical cysteine residues that have to be under a reduced state to bind their Fe-S cluster. Interestingly, the recent work that aimed at isolating TRX partners in *Chlamydomonas reinhardtii* identified several proteins belonging to the Fe-S cluster assembly machinery in chloroplasts (SUF machinery) such as NFU1/2/3, SUFB/C/D, and SUFS but also the mitochondrial NFS1 and several Fe-S proteins [65]. This may be fortuitous because these proteins have exposed and reactive cysteines when they do not bind Fe-S clusters but a control of their redox state by TRX isoforms might add a light-dependent regulation to the maturation of Fe-S proteins in chloroplasts. For instance, the reduction of an oxidized form of Arabidopsis GRXS16, a presumed SUF component, is achieved by the plastidial FTR/TRX system [66]. Hence, a TRX-dependent control of the redox state of proteins involved in Fe-S cluster biogenesis may be a unified picture among organelles and organisms.

In summary, this study might point to a connection between TRXo isoforms and the ISC assembly machinery. Although the *E. coli* ISC machinery seems able to perform the maturation of a [4Fe-4S] cluster on both Arabidopsis TRXs o, the maturation is not complete, even though these TRXs are expressed at moderate levels, at least comparable to some Fe-S proteins that are completely maturated. Evidence that the plant ISC system is also competent for this maturation and that an Fe-S cluster is present on these TRXs in a cellular context are now necessary to give more credence to a physiological role of holoforms of TRXs o. The capacity to interact with NFU4/5 and ISCA2 [22], which are the maturation factors responsible for the delivery and insertion of preformed [4Fe-4S] clusters into client proteins, is already a good indication. As the catalytic cysteines constitute the ligands of the Fe-S cluster, TRXo holoforms should not exhibit reductase activity, which was confirmed for IsTRP that is unable to reduce efficiently insulin [52]. Thus, as previously proposed for human Grx2 [68], the formation of an Fe-S cluster on TRXs o may be a convenient regulation mechanism of their reductase activity, at least for a certain pool of proteins. In the absence of a light control as for chloroplastic TRXs, having an inactive pool of labile Fe-S cluster-bridged TRXs may be a rapid and convenient way to adjust the redox metabolism during adverse conditions. In addition to other confirmed stress-responsive proteins—such as PRX IIF [7] and AOX [22]—targets that require TRXo reduction may include ISC maturation factors as NFU4/5.

## Figures and Tables

**Table 1:** Data collection and refinement statistics.

Data Collection	AtTRXo1	AtTRXo2
Beam line	FIP-BM30A	
Space group	*P*2_1_2_1_2_1_	*P*6_5_
Cell dimensions a, b, c (Å) α, β, γ (°)	37.15; 39.24; 79.34 α = β = γ = 90°	70.81; 70.81; 35.75 α = β = 90° γ = 120°
Resolution (Å)	39.67−1.80 (1.84−1.80)	35.40−1.50 (1.53−1.50)
*R*_merge_	0.122 (0.242)	0.070 (0.432)
*R*_meas_	0.131 (0.283)	0.075 (0.500)
*R*_pim_	0.049 (0.145)	0.028 (0.251)
No. unique reflections	11,316 (653)	16,473 (814)
Mean I/σI	13.1 (3.7)	20.0 (3.1)
CC_1/2_	0.996 (0.961)	0.997 (0.942)
Completeness (%)	100.0 (100.0)	99.4 (100.0)
Average redundancy	11.4 (7.0)	12.0 (7.3)
*Refinement*		
Resolution (Å)	39.67−1.80	35.40−1.50
R_free_/R_work_	23.96/21.96	17.93/16.74
Total number of atoms	1848	1882
Water	130	92
Crystallographic B-factor		
Overall B-factor (Å²)	29.16	35.36
B-factor: molecule (Å²)	28.88	35.35
B-factor: water (Å²)	32.89	35.47
*R.m.s deviations*		
Bonds	0.002	0.003
Angles	0.474	0.528
*MolProbity analysis*		
Clashscore, all atoms	2.91 (99%)	0.00 (100%)
MolProbity score	1.40 (96%)	0.50 (100%)
Protein Data Bank entry	6G61	6G62

Values in parentheses refer to data in the highest-resolution shell. AtTRXo: *Arabidopsis thaliana* thioredoxin-*o*; FIP-BM30A: French beamline for Investigation of Proteins-Bending Magnet section.

**Table 2:** Electrospray ionization mass spectrometry analysis of untreated and reduced NFU proteins

Protein	Theoretical Size (Da)	Theoretical Size without Met (Da)	Untreated	Treated with DTT	Mass Difference upon Reduction (Da)
NFU4	22,167.9	22,036.7	22,035.1	22,037.4	+2.3
NFU5	21,823.5	21,692.3	21,690.9	21,693.0	+2.1

The mass accuracy is generally ± 0.5 Da.

## Supplementary Materials

The following are available online at http://www.mdpi.com/2076-3921/7/10/142/s1, FigureS1: Purity of recombinant AtTRXo1 and AtTRXo2, Figure S2: Analytical gel filtration of *E. coli* IscS, Figure S3: Electrospray ionization mass spectrometry analysis of NFU4, Figure S4: Electrospray ionization mass spectrometry analysis of NFU5, Figure S5: The disulfide bridge of mitochondrial NFUs is reduced by NTR/TRXo system but not by NTR/GRXS15 system, Table S1: Primers used for cloning and site-directed mutagenesis experiments.
