# Supplementary material for: Mitochondrial Arabidopsis thaliana TRXo Isoforms Bind an Iron–Sulfur Cluster and Reduce NFU Proteins In Vitro
Source: Antioxidants (Basel). 2018 Oct 13;7(10):142. doi: 10.3390/antiox7100142 (PMC6210436; doi:10.3390/antiox7100142)
Supplement: Supplementary file 1 [file antioxidants-07-00142-s001.pdf]

**FigureS1. Purity of recombinant AtTRXo1 and AtTRXo2.** Sample proteins (10 µg) were separated by 15% SDS-PAGE and stained with Coomassie Brilliant Blue.

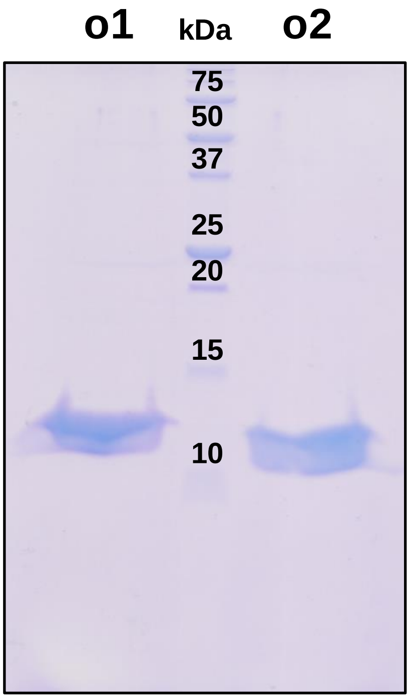


**Figure S2. Analytical gel filtration of *E. coli* IscS.** This analysis was performed by loading 100 µg of protein onto a Superdex S200 10/300 column. The presence of the polypeptide and of the PLP cofactor have been detected by the absorbance at 280 nm (blue line) and 420 nm (red line), respectively.

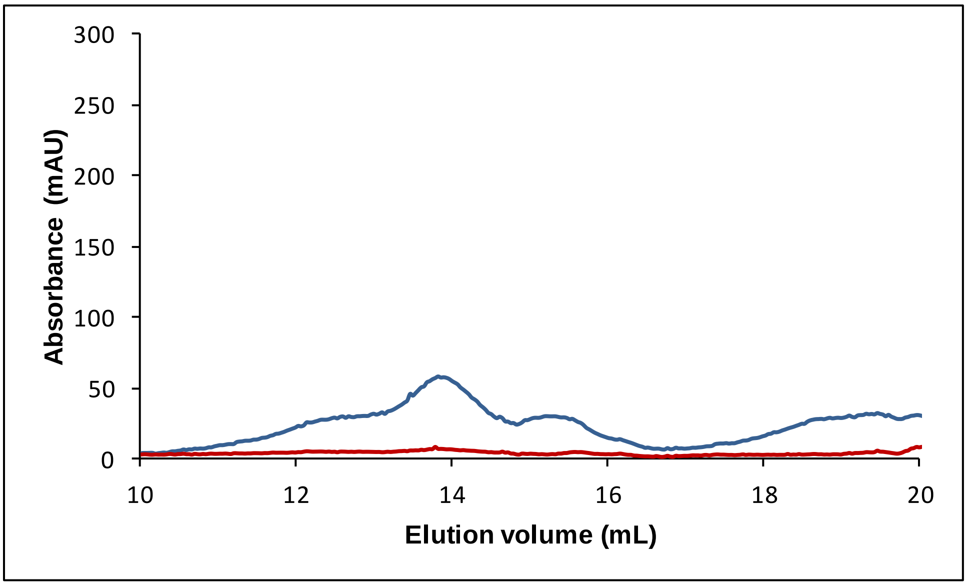

**Figure S3. Electrospray ionization mass spectrometry analysis of NFU4.** Mass spectrum of NFU4 protein determined for an untreated (A) or a reduced protein (B) as described in the "Materials and Methods" section. Species with a mass of 22,0351.1 and 22037,4 Da represent oxidized and reduced forms, respectively. Other species with sodium or Tris adduct are visible (species with a mass of 22,056.8 and 22,057.6 Da).

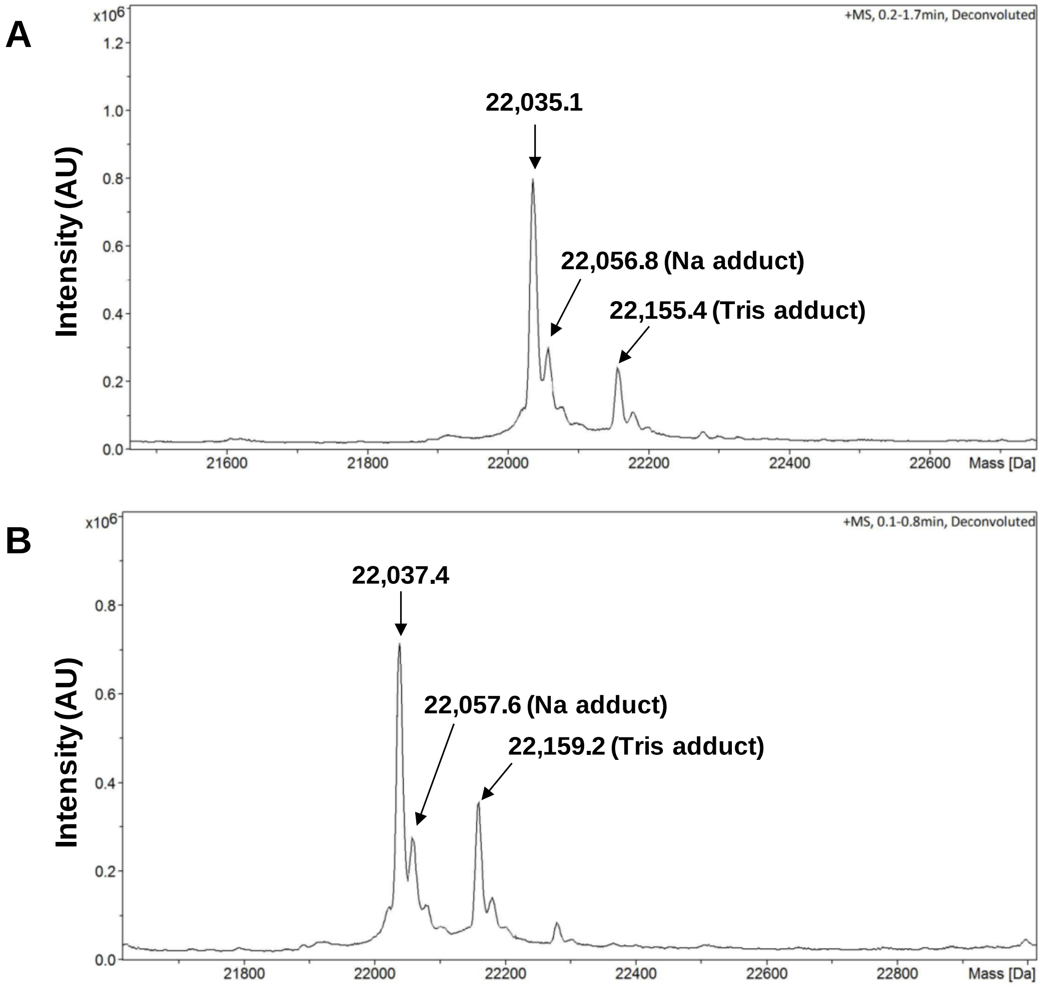

**Figure S4. Electrospray ionization mass spectrometry analysis of NFU5.** Mass spectrum of NFU5 protein determined for an untreated (A) or reduced protein (B) as described in the "Materials and Methods" section. Species with a mass of 21,690.9 and 21693.0 Da represent oxidized and reduced forms, respectively. Other species with sodium or Tris adduct are visible (species with a mass of 21,714.7 Da, 21,812.5 and 21,813.3 Da).

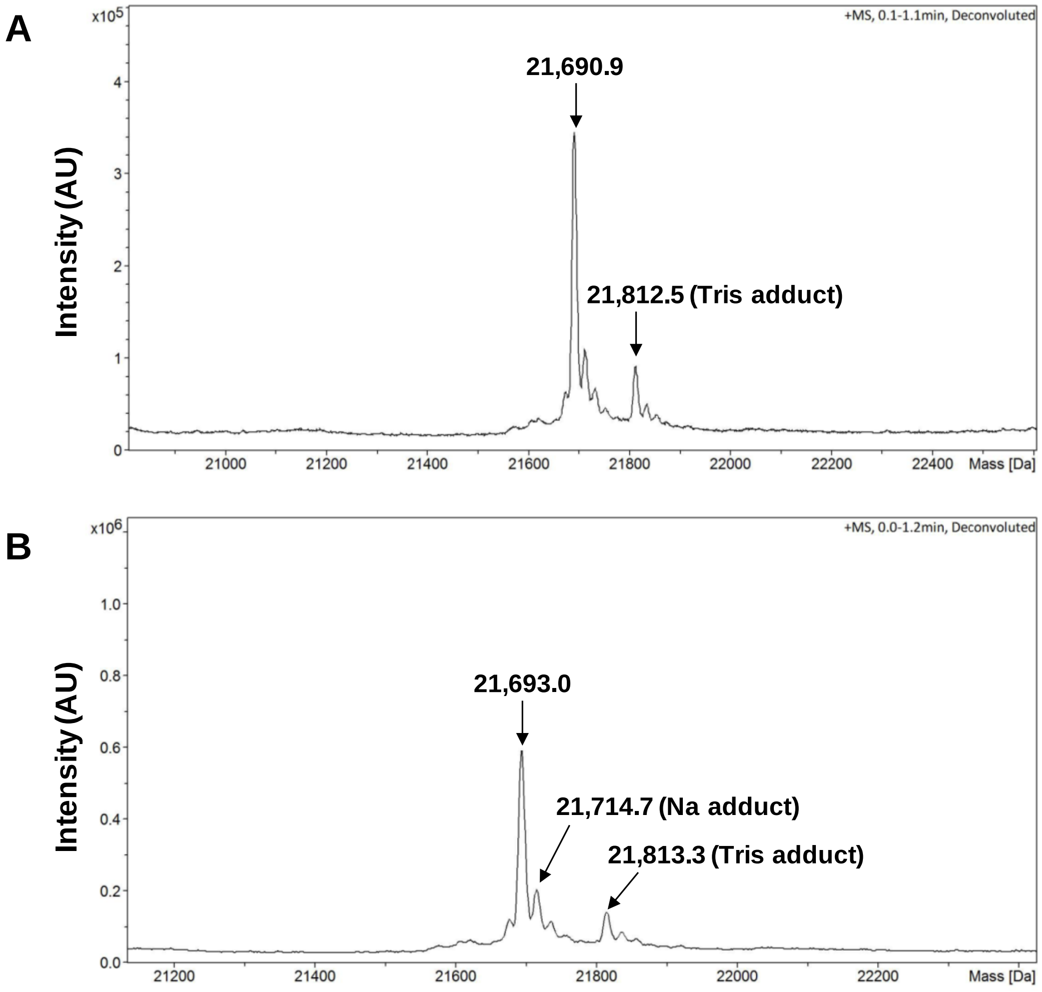

**Figure S5. The disulfide bridge of mitochondrial NFUs is reduced by NTR/TRXo system but not by NTR/GRXS15 system.** The reduction of as-purified, oxidized forms of NFU4 (A) or NFU5 (B) was assessed after a 15 min incubation in the presence of the following reducing systems: NTR: NADPH + NTR ; TRXo1: NADPH + NTR + TRXo1 ; TRXo2: NADPH + NTR + TRXo2 ; GRXS15: NADPH + NTR + GRXS15. The ability of these three oxidoreductases to reduce NFU proteins was evaluated using either 1 μM or 10 μM. After alkylation with 2 kDa mPEG maleimide, proteins were separated on non-reducing SDS-PAGE. Reduced (Red) and oxidized (Ox) proteins served as controls. The stars indicate the alkylated (*) forms of the oxidoreductases in the respective regeneration systems when visible.

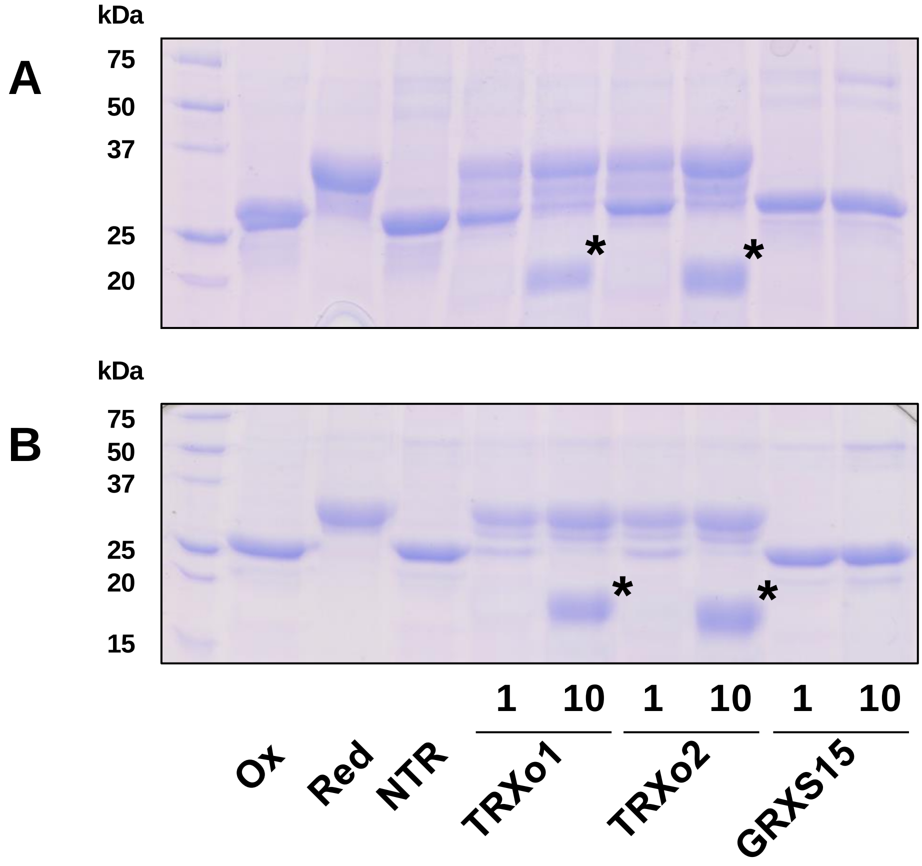

**Table S1. Primers used for cloning and site-directed mutagenesis experiments.** The *Nco*I, *Nde*I and *Bam*HI restriction sites used for cloning are underlined in the primers. The mutagenic codons are in bold.

| **Name** | **Sequence** |
|---|---|
| TRXo1 for | 5′ CCCCCCCCATATGGAAAATGGTGTTGTTCTA 3′ |
| TRXo1 rev | 5′ CCCCGGATCCTCACTTGTAGAGCTGTTC 3′ |
| TRXo2 for | 5′ CCCCCCCCATATGCGATCCAGCTTTGTAGTG 3′ |
| TRXo2 rev | 5′ CCCCGGATCCTCACTTGTAGAGTTGTTC 3′ |
| TRXo2 C37S for | 5′ TTCACTGCCGCATGG**TCT**GGACCTTGCAGGCTT 3′ |
| TRXo2 C37S rev | 5′ AAGCCTGCAAGGTCC**AGA**CCATGCGGCAGTGAA3′ |
| TRXo2 C40S for | 5′ GCATGGTGTGGACCT**AGC**AGGCTTATCTCTCCT3′ |
| TRXo2 C40S rev | 5′ AGGAGAGATAAGCCT**GCT**AGGTCCACACCATGC3′ |
| NFU4 for | 5′ CCCCCATGGCTTTTATCCAAACCCAATCA 3′ |
| NFU4 rev | 5′ CCCCGGATCCCTACTCTACTCTCATCTC 3′ |
| NFU5 for | 5′ CCCCCATGGCTTTTATCCAAACC 3′ |
| NFU5 rev | 5′ CCCCGGATCCTCACTCCATTGGACCAGA 3′ |
| IscS for | 5′ CCCCCCCCATATGTACGGAGTTTATAGAGCA 3′ |
| IscS rev | 5′ CCCCGGATCCTTAATGATGAGCCCATTC 3′ |
